# miRNA and mRNA expression analysis reveals potential sex-biased miRNA expression

**DOI:** 10.1038/srep39812

**Published:** 2017-01-03

**Authors:** Li Guo, Qiang Zhang, Xiao Ma, Jun Wang, Tingming Liang

**Affiliations:** 1 Department of Bioinformatics, School of Geographic and Biologic Information, Nanjing University of Posts and Telecommunications, Nanjing, 210023, China; 2 Department of Biostatistics, School of Public Health, Nanjing Medical University, Nanjing, 211166, China; 3 Jiangsu Key Laboratory for Molecular and Medical Biotechnology, College of Life Science, Nanjing Normal University, Nanjing, 210023, China

## Abstract

Recent studies suggest that mRNAs may be differentially expressed between males and females. This study aimed to perform expression analysis of mRNA and its main regulatory molecule, microRNA (miRNA), to discuss the potential sex-specific expression patterns using abnormal expression profiles from The Cancer Genome Atlas database. Generally, deregulated miRNAs and mRNAs had consistent expression between males and females, but some miRNAs may be oppositely expressed in specific diseases: up-regulated in one group and down-regulated in another. Studies of miRNA gene families and clusters further confirmed that these sequence or location related miRNAs might have opposing expression between sexes. The specific miRNA might have greater expression divergence across different groups, suggesting flexible expression across different individuals, especially in tumor samples. The typical analysis regardless of the sex will ignore or balance these sex-specific deregulated miRNAs. Compared with flexible miRNAs, their targets of mRNAs showed relative stable expression between males and females. These relevant results provide new insights into miRNA-mRNA interaction and sex difference.

Sex is an important factor to affect the risk of cancer occurrence and development, incidence, prognosis and treatment response, and sex-specific therapeutic strategies should be quite urgent in disease treatment. Yuan *et al*. performed a systematic molecular-level analysis (mainly including mRNA, DNA methylation, microRNA (miRNA) and protein expression levels), and suggested to develop sex-specific therapeutic strategies in certain cancer types^1^. Recent studies suggest that proteomes may differ between males and females^2^,^3^, and sex differences may exist in mRNA^4^, miRNA expression^5^,^6^,^7^ and isomiR (miRNA variants) expression levels^8^,^9^. For example, *AT2R* can influence vascular responses with sexually dimorphic^10^, and some genes are influenced by androgens or estrogen^11^,^12^. Moreover, the relationship between time and the RNA ratio is also affected by sex difference^13^. Some miRNAs differ between sexes with respect to concentration^14^,^15^, and miR-137 promoter methylation is a female-associated molecule^16^. Therefore, sex differences may exist in miRNA and mRNA profiling, and the sex-associated functions may also exist.

However, for expression differences in miRNA and mRNA between males and females, few systematic studies are performed, especially those based on miRNA-mRNA interactions. Although some of them may be influenced by difference of androgen or estrogen, understanding of the expression divergence may help us understand occurrence and development of complex diseases. Moreover, researches show that different miRNAs may have close physical distances on chromosomes within a single polycistronic transcript^17^,^18^,^19^ or have higher sequence similarity^20^,^21^,^22^,^23^. These miRNAs have close physical relationships (clustered miRNAs, miRNA gene cluster) or higher sequence similarities (homologous miRNAs, miRNA gene family), and they have close functional and evolutionary relationships^24^,^25^,^26^,^27^,^28^,^29^. Indeed, miRNA-miRNA interactions are frequent in human miRNAs^30^. Whether relevant clustered or homologous miRNAs are also diverged between males and females if expression of a specific miRNA is sexually dimorphic? Based on the important regulatory role of miRNA in the coding-non-coding RNA regulatory network, its role is achieved via targeting mRNAs to cause mRNA degradation or inhibit gene translation^31^,^32^. Then, whether target mRNA is also diverged between different sexes if miRNA is sexually dimorphic?

Thus, to understand sex differences and miRNA and mRNA expression, particularly their interactions, we selected specific and shared diseases in males and females to assess differences in miRNA and mRNA expression using public sequencing datasets, and selection of sex-specific diseases contributed to simultaneously understand miRNA and mRNA expression via comparison of shared diseases. Expression patterns of clustered or homologous miRNAs and miRNA-mRNA interactions were further discussed.

## Results

### Some deregulated miRNAs and mRNAs showed diverse expression

**Figure 1:**
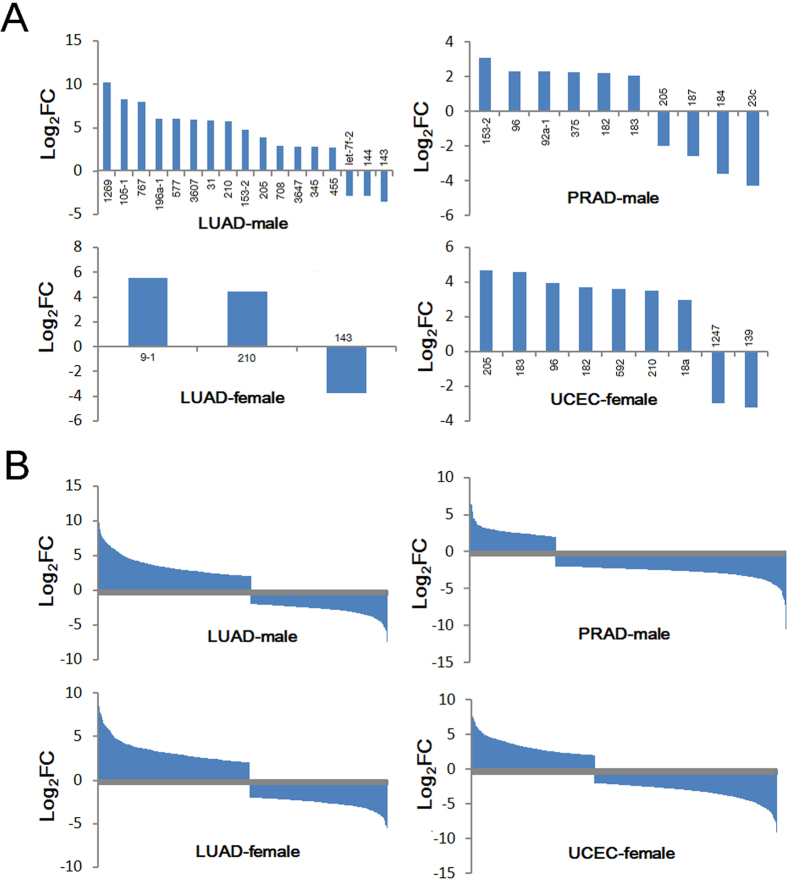
Distributions of deregulated miRNAs and mRNAs. (**A**) Distribution of deregulated miRNAs in different groups; (**B**) distribution of deregulated mRNAs in different groups.

A total of 30 deregulated miRNAs were collected in the four groups (LUAD-male, male patients with lung adenocarcinoma; LUAD-female, female patients with lung adenocarcinoma; PRAD-male, male-specific disease of prostate adenocarcinoma; UCEC-female, female-specific disease of uterine corpus endometrial carcinoma) based on strict screening criteria, and 76.67% were specifically deregulated in specific group, especially in LUAD-male group (Figure S1A). In LUAD-female group, only 3 miRNAs were identified as deregulated miRNAs. Compared to rare collected miRNAs, more mRNAs were obtained in each group, including 89 shared mRNAs in the four groups (Figure S1B). Of these, 63.04% were identified as specifically abnormally expressed mRNAs. Among of these deregulated miRNAs, up-regulated miRNAs were dominant, and only fewer miRNAs were down-regulated in relevant tumor samples (Fig. 1A). For deregulated mRNAs, down-regulated miRNAs were dominant in PRAD-male group (72.85%), but about half of mRNAs were up-regulated or down-regulated in other groups (Fig. 1B). The results shown here were in whole based upon data generated by The Cancer Genome Atlas (TCGA) Research Network: http://cancergenome.nih.gov/.

**Figure 2:**
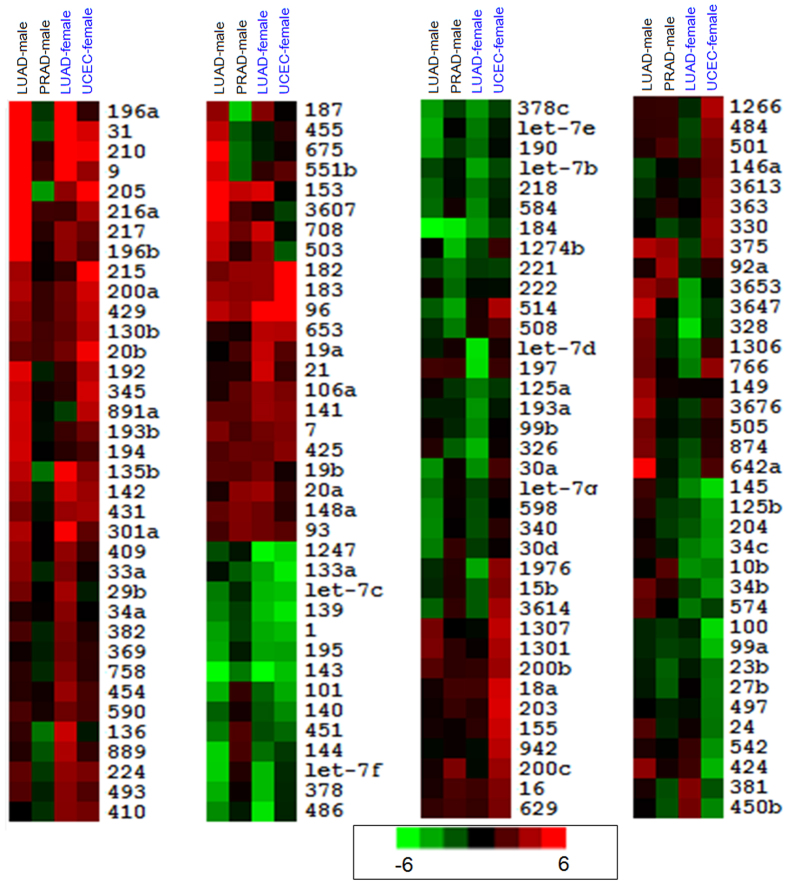
Cluster analysis of abundantly expressed miRNAs. All miRNAs were abundantly expressed, although some were not deregulated miRNAs.

**Figure 3:**
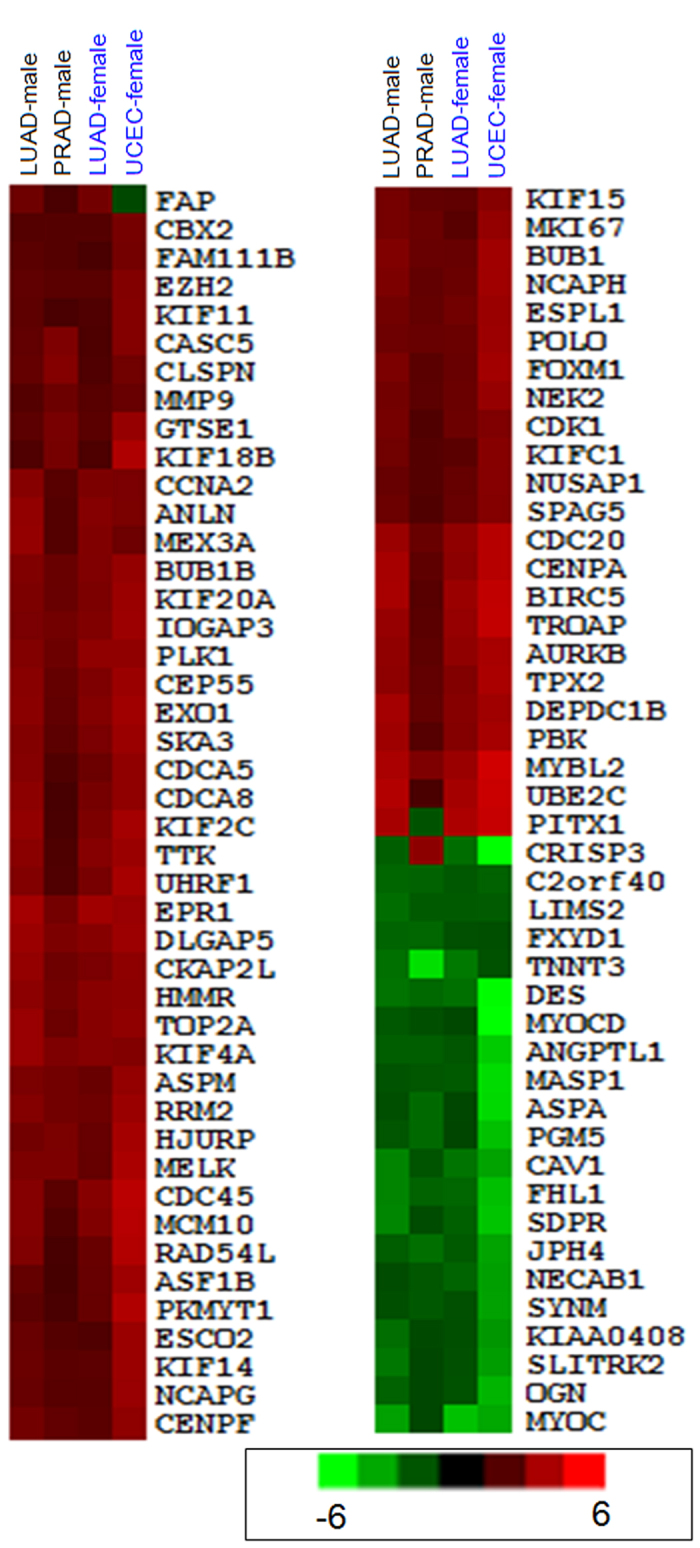
Cluster analysis of common deregulated mRNAs in four groups.

Based on abundantly expressed miRNAs, we found that most miRNAs had consistent expression across the four groups, although many had different degrees of deregulated expression in corresponding tumors (Fig. 2). For example, miR-135b was significantly up-regulated in LUAD-male, LUAD-female and UCEC-female groups (log_2_FC = 2.63, 3.50 and 1.87, respectively; FC: fold-change), but miRNA was significantly down-regulated in PRAD-male group (log_2_FC = −1.59). miR-136 and miR-187 loci were down-regulated in PRAD-male group, but over-expressed or stably expressed in other diseased samples (Fig. 2). Compared to expression divergence of miRNAs, most shared deregulated mRNAs had consistent expression patterns, and only rare mRNAs had opposing deregulated expression patterns across different groups (Fig. 3). Specifically, *CRISP3* was up-regulated in PRAD-male group and down-regulated in other groups. *FAP,* and *PITX1* also had opposite expression across the different groups as well as *CRISP3.*

**Figure 4:**
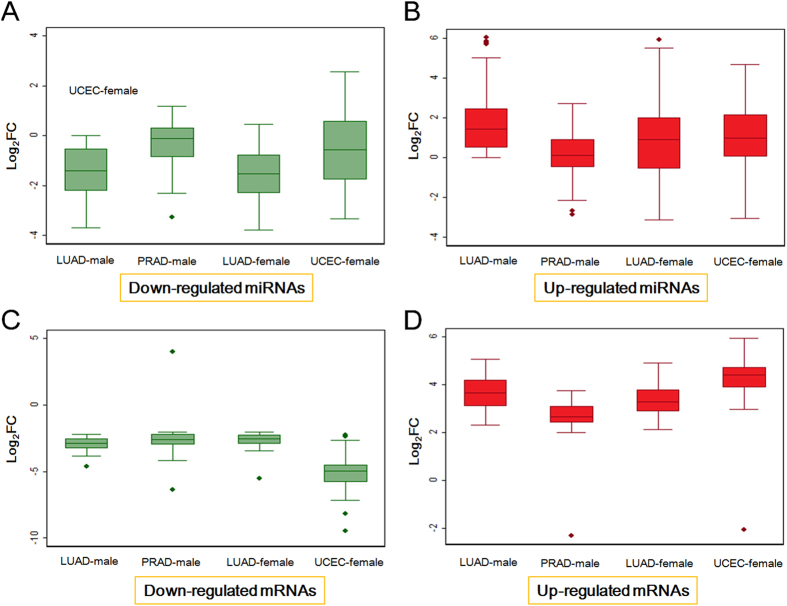
Box plots of deregulated miRNA and mRNA populations.

According to up-regulated and down-regulated miRNA and mRNA populations (Figs 3 and 4), respectively, variation was analyzed and presented using box plots. All deregulated miRNAs and mRNAs were collected from the four groups, but diverse distributions were noted (Fig. 4). Generally, deregulated populations had different centralized locations and tendencies of dispersion across the four groups, especially between different tissues, although these tissues were obtained from the same sex (Fig. 4). Distribution at the miRNA level was greater than the mRNA level, and means of log_2_FC values also fluctuated across different groups. Interestingly, the distribution of down-regulated mRNA in UCEC-female group was more dispersed than other groups (Fig. 4D).

**Figure 5:**
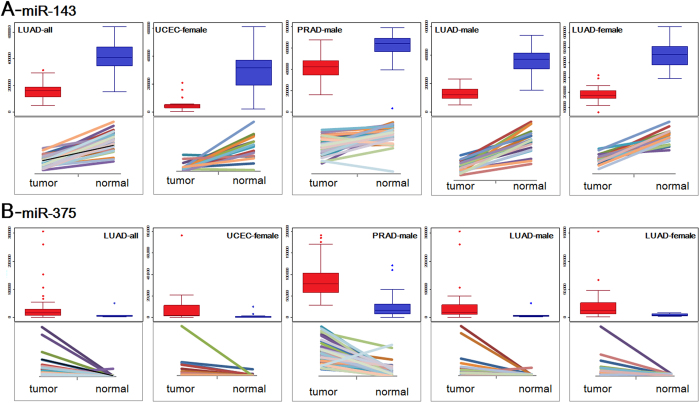
Box plots and displacement diagrams of dominantly expressed miRNAs across different groups. We selected abundantly expressed miR-143 and miR-375 for box plots and displacement based on all the paired samples in these relevant diseases and mixed samples.

Based on collected paired tumor and normal samples, we selected two quite abundant miRNAs (miR-143 and miR-375) to perform expression distribution analysis across different individuals. The two miRNAs were quite abundantly enriched in different groups (including tumor and normal tissues), and they showed down-regulated and up-regulated expression in tumor samples, respectively (Fig. 5 and Table S1). Compared with normal tissues, they showed deregulation patterns, and larger expression fluctuation could be found in tumor samples (Fig. 5). Although the two miRNAs showed consistent over-expression or low-expression, the difference levels were diverged in different groups (Table S1). For example, miR-143 showed relative expression in PRAD-male group, but it showed low-expression in other groups.

### Expression patterns of miRNA gene clusters and families

**Figure 6:**
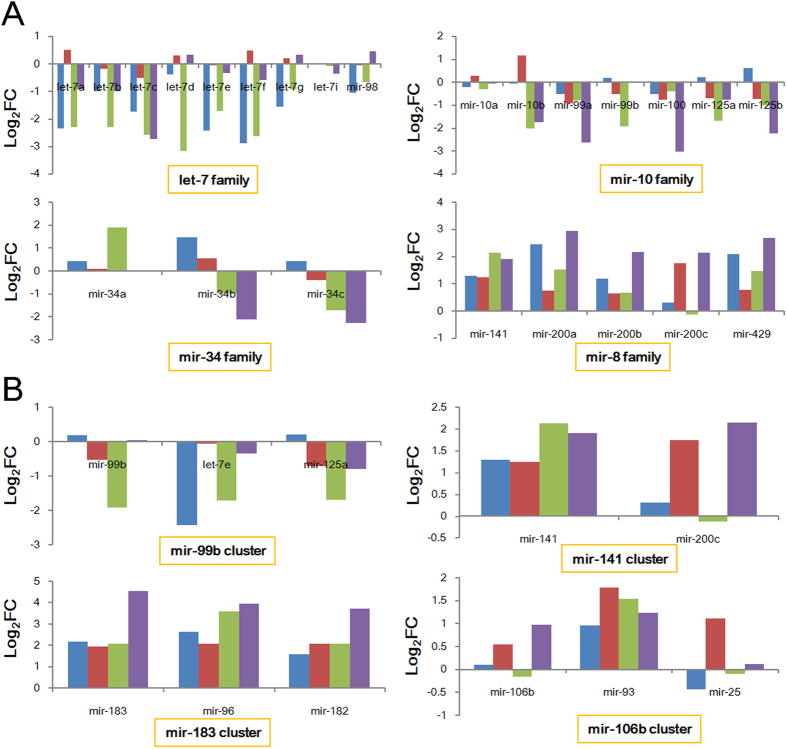
Deregulation expression patterns of miRNA gene clusters and families across different groups. Blue column: LUAD-male group; red column: PRAD-male group; green column: LUAD-female group; purple column: UCEC-female group.

Although clustered and homologous miRNAs may have functional relationships, these miRNAs had diverse degrees of deregulated expression (Fig. 6). Generally, related miRNAs had consistent deregulated expression across different groups, although they had various log_2_FC values, and some had opposing expression. For example, three members of the miR-34 gene family had diverse expression across the four groups. miR-34a was up-regulated in PRAD-male group, and was not abnormally expressed in the other groups; miR-34b and miR-34c had inconsistent expression across different groups (Fig. 6A). Expression in some clustered or homologous miRNAs was similar to single miRNA, although these miRNA gene clusters or families always involved several members.

### Functional analysis revealed potential relationships with cancer hallmark GO terms

Based on dominantly expressed miRNAs and mRNAs in these groups, especially for miRNA-mRNA interactions using experimental validation or computational prediction, integrated deregulated RNA molecules had important roles in many basic biological processes and cancer-related pathways, including regulation of the actin cytoskeleton, the Wnt signaling pathway, and melanogenesis (Table S2). According to reported cancer hallmark network framework based on sequencing data and GO term analysis^33^,^34^, some of these relevant enriched terms were consistent with the reported cancer hallmarks (such as cancer cell self-stimulate their own growth, multiply forever, invade local tissue and spread to distant organs, abnormal metabolic pathways and inflammation).

### Expression and functional analysis in the same tissues between males and females

For LUAD groups, although most miRNAs had consistent deregulated expression, some specific miRNAs had opposite expression patterns between females and males (Fig. 2). For example, miR-3647, miR-328 and miR-1306 had opposing expression between male and female samples. At the mRNA level, according to shared deregulated mRNAs among the four groups, all mRNAs were consistently expressed in the same male and female tissues (Fig. 3). Moreover, based on abnormally mRNA expression in male and female, no mRNA was detected with opposing deregulated expression. Further analysis showed that rare mRNAs had similar diverged expression compared to miRNA.

**Figure 7:**
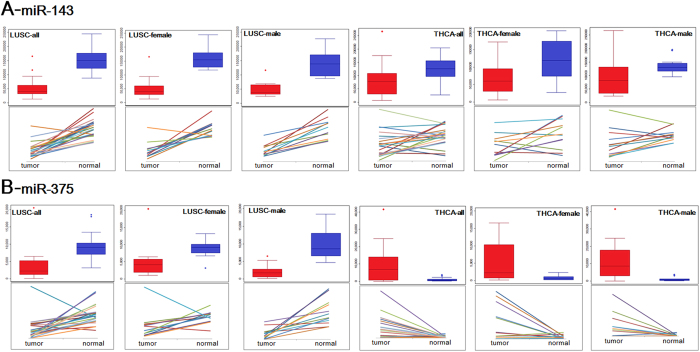
Expression distributions of miR-143 and miR-375 in LUSC and THCA. The two miRNAs were analyzed in paired samples, and mixed samples were also analyzed to understand the expression profiles across different samples.

Box plots based on deregulated miRNA and mRNA populations showed that up-regulated miRNA were prone to dispersion (Fig. 4A). Other down-regulated miRNAs and deregulated mRNAs were similarly distributed between male and female tissues including the means and inter-quartile range (Fig. 4). Moreover, based on dominantly expressed miR-143 and miR-375, using LUSC (Lung squamous cell carcinoma) and THCA (Thyroid carcinoma) samples (Fig. 7), we noted divergence of miR-143 occurred in THCA between males and females (Fig. 7A).

## Discussion

Non-coding RNAs (ncRNAs) are of interest because of their versatile roles in biological and pathological processes, particularly widely studied small miRNAs and long non-coding RNAs (lncRNAs). miRNA-disease association studies have reported in the context of potential roles in diseases via their expression and function^35^,^36^,^37^,^38^,^39^,^40^, and predictions of associations may help reveal roles of miRNAs in coding-non-coding RNA network and occurrence and development of diseases. Relationships between ncRNA and disease may also be useful for biomedical studies 41,42,43,44, and the sex-based differences may be important. Specifically, estrogen may play a role in disease incidence, such as adenocarcinomas between females and males^45^. Sex-related differences may cause abnormally expressed miRNA and mRNA although few studies have documented these dimorphisms. Thus, with data from patients and volunteers, we selected homogeneous female and male samples and found that mRNAs are prone to be down-regulated, whereas miRNAs were over-expressed in some groups (Fig. 1). miRNA and mRNA interactions are interest because expression divergence between miRNA and mRNA may be influenced by these interactions and this will contribute to understanding disease. Functional analyses using deregulated RNA molecules indicate relevant cancer hallmark network^33^,^34^, suggesting their crucial roles in pathological process.

Simon’s group reported that 54 human platelet mRNAs and 9 miRNAs were differently expressed with respect to sex^46^, and that the relevant miRNA-mRNA regulatory network may be associated with sex. Similar studies for miRNA expression have also been reported in male and female samples^47^,^48^,^49^, and relationships of miRNA expression and sex differences have attracted the attention of researchers. Herein, for the specific disease, the two groups with gender-difference, LUAD-male and LUAD-female groups, showed inconsistent miRNA and mRNA expression patterns and fewer common RNAs (Figs 1, 2, 3 and S1). Some miRNAs may show opposite deregulation expression (such as miR-328 and miR-1306), while at the mRNA levels, according to differentially expressed mRNAs in males and females, no mRNAs are detected to do this based on sex using strict filter criteria. Indeed, some mRNAs may have inconsistent expression but mRNA expression is relatively more stable than miRNA, although some specific mRNAs are involved in different levels of deregulated expression in diseased male and female samples. As a class of small non-coding RNA molecules, miRNAs, are flexible and not specific for target mRNA, which may lead to diverse expression. These differentially expressed miRNAs have important roles in basic biological processes and contribute to occurrence and development of disease. For example, miR-328, may be a potential indicator for acute myocardial infarction and is associated with increased risk of mortality^50^, and it can target CD44 expression resulting in tumor progression by enhancing ROS defenses^51^. General analysis with mixed samples may ignore or balance differentially expressed miRNAs by sex and then tracking these crucial miRNAs become impossible. Our data suggest that these miRNAs should be scrutinized further for differences between men and women.

Moreover, according to clustered and homologous miRNAs in the human genome^30^, expression and integrated analysis indicate that these location and sequence-related miRNAs have consistent deregulated expression in different groups. Some miRNA may be normally expressed in diseased samples, and other relevant miRNAs may be always simultaneously up-regulated or down-regulated despite their diverged fold-change values (Fig. 6). Similar expression patterns may contribute to co-regulation of target mRNAs^52^. Based on sex difference, some miRNA families also had expression divergence, such as miR-10 family (miR-10b, miR-99b and miR-125a) and miR-34 family (miR-34a and miR-34c), and these homologous members have opposing deregulated expression in male and female patients (Fig. 6). According to expression diversity in homologous miRNAs and clustered miRNAs, it is difficult to document that related miRNAs have consistent expression and enrichment levels, and a specific miRNA may be not expressed at certain times in certain tissues. Expression divergence of location or sequence-related miRNAs between samples from men and women may be crucial miRNAs, and these deserve more investigation. Ignoring sex differences can give the appearance of balanced or normally expressed results, so potentially crucial miRNA gene clusters or families will be missed or will not further selected and studied, especially for functions and roles in disease occurrence and development. More importantly, although some miRNAs have divergent expression in male and female, mRNA expression is relative stable or had only slightly divergent expression. miRNA-mRNA interactions may be more flexible and complex due to dynamic expression of miRNA and relative stably expressed target mRNA, particularly in direct and indirect miRNA-miRNA interactions in the coding-non-coding RNA regulatory network^30^,^52^.

Thus, some miRNAs, especially for some location- and sequence-related miRNAs, may have divergent expression between male and female samples. Compared with flexible expression of miRNAs, mRNA expression is relatively stable with respect to sex and these results complicate miRNA-mRNA and miRNA-miRNA interactions. We hope that future studies will focus on these dimorphic expression patterns and correlate these with disease.

## Materials and Methods

### Source data

All available high-throughput small RNA sequencing datasets were obtained from The Cancer Genome Atlas pilot project established by the NCI and NHGRI (http://cancergenome.nih.gov/). These datasets included small RNA and mRNA sequencing of tumor and control samples from women-specific disease (uterine corpus endometrial carcinoma, UCEC), tumor and control samples from men-specific disease (prostate adenocarcinoma, PRAD), and tumor and control samples from women and men respectively (lung adenocarcinoma, LUAD). Therefore, there were four groups with sex differences as follows: LUAD-male, LUAD-female, PRAD-male and UCEC-female. All data were sequenced using an Illumina Hiseq sequencing platform, and data had been preliminarily analyzed and annotated in the TCGA database (level 3). With respect to homogeneity (main indices included gender, race, ethnicity, etc), we selected tumor and normal samples in LUAD-male (n = 15), PRAD-male (n = 15), LUAD-female (n = 15), and UCEC-female due to limited data (n = 13). Moreover, in order to further understand expression profiles, we also performed analysis in males and females from LUSC (Lung squamous cell carcinoma) and THCA (Thyroid carcinoma) (n = 12 in the two diseases). Furthermore, according to preliminary analysis, we selected some abundant miRNAs to perform further expression analysis using paired tumor and normal samples (Table S3).

### Expression and function analysis

Differentially expressed miRNAs and mRNAs were analyzed and obtained using DESeq package^53^, and abundantly and abnormally expressed mRNAs and miRNAs with statistical difference and biological difference were collected to perform further analysis (Figure S2). First, miRNA and mRNA were analyzed at miRNA and mRNA levels, respectively, and miRNA analysis was specially analyzed based on clustered and/or homologous miRNAs according to annotations in miRBase database (http://www.mirbase.org/). Then, analysis of miRNA-mRNA interactions was performed with attention to sex-base differences (Figure S2). Specifically, fewer miRNAs were selected based on strict screening criteria than mRNAs, more miRNAs may be involved in further analysis using less strict criteria, including |log_2_(FC, fold change)| ≥ 1.5. Even those miRNAs were obtained with larger fold-changes but they were not statistically different. To better understand expression patterns of these sequence- or location-related miRNAs between males and females, expression patterns of abundantly expressed miRNA gene clusters and families were analyzed across different groups.

For common differentially expressed mRNAs in four groups, cluster analysis was performed with Cluster 3.0 and TreeView 1.60 programs^54^,^55^ (http://rana.lbl.gov/eisen). Also, abundantly expressed miRNAs were also analyzed because fewer miRNAs were identified as deregulated miRNAs. Functional analysis was performed using public platform, CapitalBio Molecule Annotation System V4.0 (MAS, http://bioinfo.capitalbio.com/mas3/) according to integrated deregulated mRNAs identified as target mRNAs of deregulated miRNAs. Target mRNAs of crucial miRNAs were collected from the public miRTarBase database^56^, and these target mRNAs were identified with experimental methods. If few or no targets were reported in the database, predicted targets were obtained from the TargetScan program^57^. For abnormal gene and miRNA expression in tumors, we also checked the cancer hallmark GO terms^33^,^34^.

### Statistical analysis

miRNA and mRNA relative expression levels are depicted as means ± SD (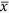 ± SD) and differentially expressed RNAs were obtained with DESeq software if *P*_adj_ values < 0.05 and the |log_2_(FC)| ≥ 2.0. Venny’s distribution was analyzed with Venny web Server 2.0 (http://bioinfogp.cnb.csic.es/tools/venny/index.html) to understand distributions across different groups. To understand distributions of abnormally expressed miRNAs and mRNAs, characteristics of distribution were analyzed based on down-regulated and up-regulated miRNA and mRNA populations, respectively. Paired *t*-test was used to estimate the potential expression divergence between paired tumor and normal samples. All the relevant statistical analysis was performed using STATA software (version 13.0), and if *P* or *P*_adj_ < 0.05, the difference was considered to be statistically significant.

## Additional Information

**How to cite this article**: Guo, L. *et al*. miRNA and mRNA expression analysis reveals potential sex-biased miRNA expression. *Sci. Rep.* **7**, 39812; doi: 10.1038/srep39812 (2017).

**Publisher's note:** Springer Nature remains neutral with regard to jurisdictional claims in published maps and institutional affiliations.

## Supplementary Material

Supplementary Information
