# Supplementary material for: miRNA and mRNA expression analysis reveals potential sex-biased miRNA expression
Source: Sci Rep. 2017 Jan 3;7:39812. doi: 10.1038/srep39812 (PMC5206641; doi:10.1038/srep39812)
Supplement: Supplementary Information [file srep39812-s1.pdf]

miRNA and mRNA expression analysis reveals potential sex-biased miRNA expression

Li Guo, Qiang Zhang, Xiao Ma, Jun Wang, Tingming Liang

Table S1: Statistical analysis based on the data in Figure 6.

miR-143	Log_2(FC)	*t*	*P*	miR-375	Log_2(FC)	*t*	*P*
Luad-all (n=41)	-1.36	-16.5429	0.0001	Luad-all (n=41)	2.56	3.1227	0.0033
Ucec-female (n=18)	-2.35	-6.4633	0.0001	Ucec- female (n=18)	3.11	2.0372	0.0575
Prad-male (n=53)	-0.54	-9.7859	0.0001	Prad-male (n=53)	1.92	10.2413	0.0001
Luad-male (n=21)	-1.49	-11.3897	0.0001	Luad-male (n=21)	2.69	2.3472	0.0293
Luad-female (n=20)	-1.27	-12.0082	0.0001	Luad-female (n=20)	2.31	2.6060	0.0174

Table S2: Functional analysis based on deregulated miRNAs and mRNAs.

Pathway	P-value
Regulation of actin cytoskeleton	9.86E-06
Wnt signaling pathway	9.10E-07
Melanogenesis	6.84E-08
MAPK signaling pathway	3.54E-04
Tight junction	7.18E-06
Focal adhesion	6.74E-04
Axon guidance	8.21E-05
Calcium signaling pathway	0.003555
Alzheimer's disease	0.003096
Purine metabolism	0.001786

The table only lists the top 10 enriched pathways.

Table S3: Selected paired tumor and normal samples in the three diseases.

Diseases	Tumor	Normal	Total
LUAD	41	41	82
UCEC	18	18	36
PRAD	53	53	106
LUAD-male	21	21	42
LUAD-female	20	20	40

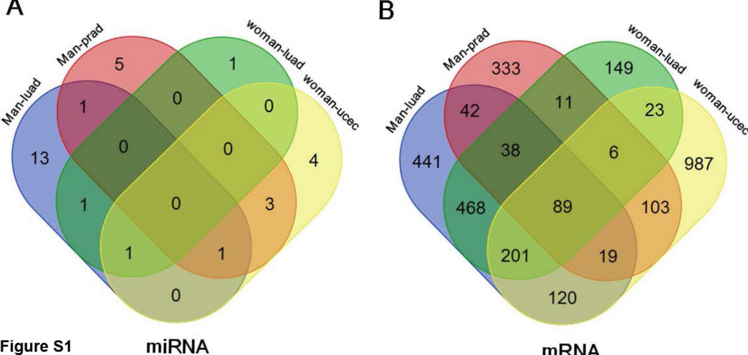
A
Man-luad
Man-prad
woman-luad
woman-ucec
5
1
13
1
0
0
4
0
0
1
0
3
1
1
0
miRNA
B
Man-luad
Man-prad
woman-luad
woman-ucec
333
149
42
11
23
441
38
6
987
468
89
103
201
19
120
mRNA

Figure S1

Figure S2

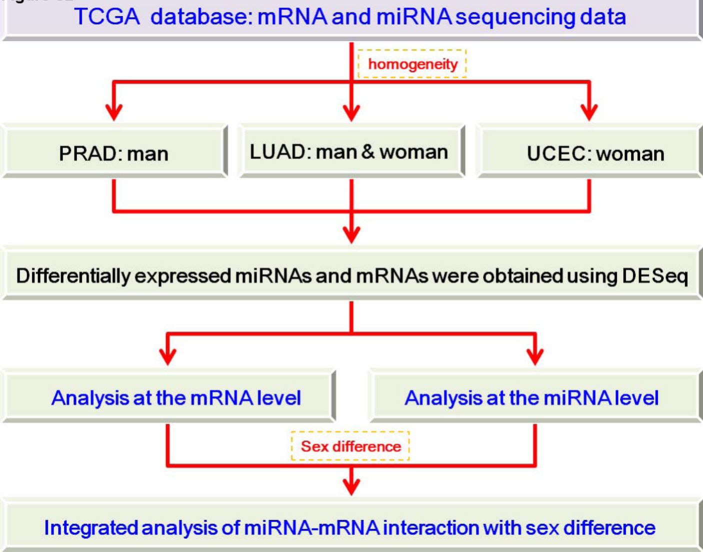
TCGA database: mRNA and miRNA sequencing data
homogeneity
PRAD: man
LUAD: man & woman
UCEC: woman
Differentially expressed miRNAs and mRNAs were obtained using DESeq
Analysis at the mRNA level
Analysis at the miRNA level
Sex difference
Integrated analysis of miRNA-mRNA interaction with sex difference
